# Use of Outpatient Rehabilitation Among Adult Stroke Survivors — 20 States and the District of Columbia, 2013, and Four States, 2015

**DOI:** 10.15585/mmwr.mm6720a2

**Published:** 2018-05-25

**Authors:** Carma Ayala, Jing Fang, Cecily Luncheon, Sallyann Coleman King, Tiffany Chang, Matthew Ritchey, Fleetwood Loustalot

**Affiliations:** 1 Division for Heart Disease and Stroke Prevention, National Center for Chronic Disease Prevention and Health Promotion, CDC.

Stroke is a leading cause of mortality and disability in the United States (*1*,*2*). Approximately 800,000 American adults experience a stroke each year (*2*,*3*). Currently, approximately 6 million stroke survivors live in the United States (*2*). Participation in stroke rehabilitation (rehab), which occurs in diverse settings (i.e., in-hospital, postacute care, and outpatient settings), has been determined to reduce stroke recurrence and improve functional outcomes and quality of life (*3*,*4*). Despite longstanding national guidelines recommending stroke rehab, it remains underutilized, especially in the outpatient setting. Professional associations and evidence-based guidelines support the increasing stroke rehab use in health systems and are promoted by the public health community (*3*–*6*). An analysis of 2005 Behavioral Risk Factor Surveillance System (BRFSS) data revealed that 30.7% of stroke survivors reported participation in outpatient rehab for stroke after hospital discharge in 21 states and the District of Columbia (DC) (*7*). To update these estimates, 2013 and 2015 BRFSS data were analyzed to assess outpatient rehab use among adult stroke survivors. Overall, outpatient rehab use was 31.2% (20 states and DC) in 2013 and 35.5% (four states) in 2015. Disparities were evident by sex, race, Hispanic origin, and level of education. Focused attention on system-level interventions that ensure participation is needed, especially among disparate populations with lower levels of participation.

BRFSS is a telephone survey of the noninstitutionalized U.S. population* conducted annually by all states. The cardiovascular health module, which includes questions about rehab participation, was an optional module in 2013 and 2015. In 2013, the median cardiovascular health module response rate^†^ for 20 states^§^ was 46.2%. Among the four states^¶^ participating in the module in both 2013 and 2015, the response rate was 49.3% in 2013 and 51.5% in 2015.

Stroke survivors were identified by the question “Has a doctor, nurse, or other health professional ever told you that you had a stroke?” Participation in outpatient stroke rehab was only asked of those with a history of stroke and was identified among respondents who answered “yes” to the question “Following your stroke, did you go to any kind of outpatient rehabilitation?” Demographic characteristics collected included age, sex, race, Hispanic origin, education (less than high school, high school graduate, some college, or college graduate) and health insurance status. Selected cardiovascular disease risk factors included hypertension, high blood cholesterol, diabetes, obesity, and current smoking. Percentages of respondents who participated in stroke rehab were measured, overall, by demographic characteristics, by cardiovascular disease risk factors in 2013, and by state of residence, and were adjusted for age, sex, race/Hispanic origin, education, insurance status, presence of cardiovascular disease risk factors, and number of cardiovascular disease risk factors (0, 1, 2, 3, 4, or 5). Adjusted percentages and 95% confidence intervals (CIs) were calculated; p-values <0.05 (obtained using Wald F test) were regarded as statistically significant. Statistical software was used to account for the complex sampling design.

In 2013, among 168,655 BRFSS participants, 3.3% (95% CI = 3.1%–3.4%) reported a history of stroke and were classified as stroke survivors. In 2015, among 21,047 participants, 3.3% (95% CI = 3.0%–3.8%) were stroke survivors. In 2013, stroke outpatient rehab participation was 31.2% (95% CI = 29.1%–33.4%) (Table 1). Men, non-Hispanic blacks, and those with a college education or higher more frequently reported participating in stroke outpatient rehab than did women, non-Hispanic others, Hispanics, and those with less than a high school education.

**TABLE 1:** Unadjusted and adjusted* percentages of adults who survived a stroke and received outpatient stroke rehabilitation, by demographic characteristics and presence of cardiovascular disease risk factors — Behavioral Risk Factor Surveillance System (BRFSS), 20 U.S. states and the District of Columbia, 2013

Characteristic	Sample size	Unadjusted % (95% CI)	P-value^ꝉ^	Adjusted* % (95% CI)	P-value
**Total**	**6,743**	**31.2 (29.1–33.4)**	**—**	**31.2 (29.1–33.4)**	**<0.001**
**Sex**					
Men	2,616	33.7 (30.4–37.2)	0.038	33.8 (30.5–37.2)	0.030
Women	4,127	29.1 (26.5–31.9)		29.1 (26.4–31.8)	
**Age group (yrs)**					
18–64	2,468	30.9 (27.6–34.4)	0.798	30.6 (27.5–34.0)	0.622
≥65	4,275	31.5 (29.0–34.1)		31.7 (29.0–34.6)	
**Race/Ethnicity**					
White, non-Hispanic	5,132	30.3 (28.0–32.7)	0.019	30.0 (27.6–32.5)	0.013
Black, non-Hispanic	966	39.1 (32.9–45.7)		39.8 (33.6–46.2)	
Hispanic	481	26.5 (20.0–34.1)		26.7 (20.3–34.3)	
Other, non-Hispanic	164	24.4 (15.9–35.6)	—^§^	25.8 (17.0–37.0)	—^§^
**Education**					
Less than high school	1,141	25.8 (21.5–30.6)	0.025	25.7 (21.4–30.5)	0.022
High school	2,255	32.5 (28.9–36.2)		32.3 (28.8–36.2)	
Some college	1,923	31.9 (28.0–36.1)		32.1 (28.3–36.2)	
College or higher	1,424	36.3 (31.5–41.2)		36.4 (31.5–41.5)	
**Insurance**					
Yes	6,276	31.6 (29.5–33.7)	0.492	31.3 (29.2–33.5)	0.836
No	467	28.2 (20.2–38.0)		30.3 (21.9–40.2)	
**CVD risk factors (no.)^¶^**					
0	455	35.6 (27.0–45.3)	0.382	35.6 (27.4–40.2)	0.478
1	1,349	29.2 (24.7–34.2)		29.4 (24.9–44.7)	
2	2,103	30.1 (26.5–33.9)		30.3 (26.7–34.4)	
3	1,805	31.5 (28.0–35.2)		31.2 (27.7–34.9)	
4	918	34.8 (28.7–41.5)		34.5 (28.5–41.1)	
5	113	21.8 (12.6–35.1)	—^§^	22.9 (13.3–36.5)	—^§^

**Abbreviations:** CI = confidence interval; CVD = cardiovascular disease.

* Adjusted for age, sex, race/ethnicity, education, insurance status, and CVD risk.

^ꝉ^ P-values were obtained using Wald F test to identify statistically significant differences among subgroup.

^§^ BRFSS recommends that data be suppressed when relative standard error (RSE) is >30% or denominator <50; it is also suggested that if RSE is 20%–30%, the estimates are potentially unreliable.

^¶^ Selected self-reported CVD risk factors include hypertension, high blood cholesterol, diabetes, obesity, and current smoking. Categories were assigned based on the number of risk factors present: 0, 1, 2, 3, 4, or 5.

Total adjusted outpatient rehab participation was 31.2% in 2013 and 35.5% in 2015 (Table 2). In 2013, adjusted percentages ranged from 23.1% in Oregon to 43.6% in Minnesota. The unadjusted and adjusted percentages of stroke survivors who took part in outpatient rehab in 2015 were lowest in Maine (28.0% and 31.3%, respectively) and highest in Iowa (46.1% and 49.8%, respectively). Among the four states that included stroke outpatient rehab questions in both 2013 and 2015, the overall adjusted percentage of stroke outpatient rehab participation increased 8.3 percentage points, from 27.2% in 2013 to 35.5% in 2015 (p*<*0.05).

**TABLE 2:** Crude and adjusted percentages* of adults who survived a stroke and received stroke outpatient rehabilitation, by state and ascending adjusted percentage — Behavioral Risk Factor Surveillance System, 20 U.S. states and the District of Columbia (DC), 2013, and four U.S. states, 2015

Year/State^ꝉ^	No.	Unadjusted % (95% CI)	P–value^§^	Adjusted* % (95% CI)	P–value^§^
**2013**					
Total (20 states and DC)	6,743	31.2 (29.1–33.4)	0.003	31.2 (29.1–33.4)	0.012
Oregon	202	22.7 (16.8–29.9)		23.1 (17.1–30.5)	
Georgia	306	24.8 (19.3–31.2)		23.7 (18.5–29.9)	
Oklahoma	197	23.8 (17.0–32.3)		24.6 (17.3–33.5)	
Hawaii	210	24.1 (17.4–32.3)		25.9 (17.8–36.0)	
North Dakota	230	26.0 (19.5–33.8)		27.0 (20.3–34.9)	
Maine	139	27.2 (19.4–36.6)		28.1 (20.2–37.6)	
Tennessee	254	27.7 (20.8–35.9)		28.1 (21.0–36.5)	
Arkansas	278	27.9 (20.9–36.2)		29.1 (22.3–37.0)	
Florida	1,618	30.6 (25.8–35.8)		30.0 (25.4–35.0)	
Washington	378	30.6 (24.5–37.5)		30.8 (24.5–37.9)	
Arizona	170	30.7 (20.9–42.6)		32.1 (22.2–43.9)	
Wisconsin	166	31.1 (21.2–42.9)		32.4 (22.3–44.4)	
District of Columbia	162	39.0 (28.5–50.8)		32.7 (22.5–44.8)	
Missouri	319	31.8 (24.8–39.7)		32.8 (25.8–40.7)	
North Carolina	204	32.9 (24.5–42.6)		33.4 (24.8–43.2)	
Mississippi	426	35.5 (28.4–43.2)		34.0 (26.8–42.0)	
South Carolina	441	37.4 (31.1–44.1)		35.8 (29.6–42.5)	
Massachusetts	112	38.9 (24.1–56.0)	—^¶^	37.8 (24.8–52.9)	—^¶^
Nebraska	291	38.9 (30.6–47.8)		39.6 (30.7–49.3)	
Iowa	263	40.6 (33.4–48.1)		41.7 (34.5–49.4)	
Minnesota	377	43.0 (31.5–55.2)		43.6 (31.0–57.1)	
**2013**					
Total (four states)	910	27.2 (23.5–31.3)	0.001	27.4 (23.5–31.3)	0.0004
Oregon	202	22.7 (16.8–29.9)		22.7 (16.2–30.8)	
Georgia	306	24.8 (19.3–31.2)		24.2 (18.8–30.6)	
Maine	139	27.2 (19.4–36.6)		28.4 (20.3–38.2)	
Iowa	263	40.6 (33.4–48.1)		41.7 (34.4–49.4)	
**2015**					
Total (four states)	729	35.5 (29.6–41.8)	0.033	35.5 (29.6–41.8)	0.008
Maine	182	28.0 (21.0–36.3)		31.3 (23.1–41.0)	
Georgia	201	34.0 (25.4–43.8)		31.8 (24.2–40.6)	
Oregon	180	36.2 (26.8–46.7)		39.7 (29.6–50.8)	
Iowa	166	46.1 (37.2–55.3)		49.8 (40.3–59.3)	

**Abbreviation:** CI = confidence interval.

* Adjusted for age, sex, race/ethnicity, education, insurance status and cardiovascular disease risk factors.

^ꝉ^ States are listed in ascending order of adjusted percentages for outpatient stroke rehabilitation in 2013 and 2015.

^§^ P-values were obtained using Wald F test to identify statistically significant differences among states within each year; comparison of differences between 2013 and 2015 among four states only was p = 0.0289.

## Discussion

Overall, approximately one third of stroke survivors reported participating in stroke outpatient rehab. Although outpatient rehab use increased significantly in the four states that collected data in both 2013 and 2015, it remained suboptimal (*3*), highlighting missed opportunities to reach stroke survivors. Stroke recovery can be a long and complex process, involving multiple domains of therapy (e.g., physical, occupational, communication, and cognitive) and occurs in inpatient rehabilitation facilities, skilled nursing facilities, and outpatient rehabilitation facilities. Benefits of stroke outpatient rehab have been determined to improve patient functional status, survival, cardiovascular risk profiles, and quality of life and reduce risks for recurrent strokes and psychological or stress disorders (*3*,*4*,*8*,*9*). Generally, stroke outpatient rehab participation is underutilized (*3*,*8*), which this study found to be true for all subgroups and states included in the analysis. No subgroup had outpatient rehab use rates >40%, and no state had use rates >50%. Although the overall prevalence of outpatient rehab use was low, disparities in use were evident. Younger adults, women, non-Hispanic persons of other than black or white races, Hispanics, and adults with less than a high school education were less likely to use stroke outpatient rehab than their counterparts. Disparities in stroke outpatient rehab at the state level were also apparent. For example, adjusted outpatient rehab use prevalence in Minnesota (43.6%) was almost twice that in Oregon (23.1%).

Increasing participation in stroke outpatient rehab has been recognized as a national priority. Healthy People 2020** aims to increase the proportion of adult stroke survivors who are appropriately and effectively assessed and referred for rehabilitation services. The estimates from the *Healthy People 2020* objective are high (90% during 2008–2011); however, they are reflective of assessment or referral, not participation (*4*). Improving coordination of care to support assessment, referral, and, ultimately, participation in rehab is needed. The continued underutilization of outpatient stroke rehab might be related to lack of patient access to outpatient facilities, ineffective referral from health care providers, high out-of-pocket costs, lack of health insurance coverage, or lack of knowledge and awareness of benefits of outpatient rehab for stroke survivors (*4*,*6*). The CDC-supported Paul Coverdell National Acute Stroke Program^††^ seeks to better understand the care provided to stroke survivors to identify disparities and support quality improvement around the assessment for, effective referral to, and provision of stroke rehab services. Experiences from such programs can support system-level changes that encourage use of stroke rehab services across all subgroups and geographies.

The findings in this report are subject to at least five limitations. First, BRFSS data are self-reported and subject to recall bias. Moreover, recall bias might lead to participants inaccurately reporting the type of stroke rehab they used (i.e., outpatient rehab versus inpatient rehabilitation facilities, skilled nursing facilities, and home health rehab) (*10*). Second, the survey does not capture stroke severity, variations in rehabilitation needs, or information about why participants did not participate in outpatient rehab. Third, the optional module was only used by selected states, and the findings should not be considered as nationally representative. Fourth, with few respondents reporting a history of stroke (162 in the District of Columbia to 1,618 in Florida), some state-level confidence intervals were wide, and results should be interpreted with caution. Finally, only participation in outpatient rehab was included in the module, limiting the ability to assess participation in other rehab modalities.

Although estimates of stroke outpatient rehab referral might be high, participation in stroke outpatient rehab remains suboptimal. Barriers to participation in stroke outpatient rehab are evident (*3*,*8*–*10*), but focused attention on system-level interventions that ensure participation is needed, especially among populations with lower levels of participation. Interventions that might improve outpatient rehab participation include increasing coverage for outpatient rehab services by health insurers, reducing copayments, extending rehab clinic hours to improve access availability, and implementing standardized assessments by health care professionals to guide appropriate referrals to outpatient rehab at hospital discharge (*3*–*5*,*8*). Stroke survivors should be educated about stroke outpatient rehab opportunities possibly available in their community that reduce barriers related to transportation and time (e.g., telehealth, mobileHealth, and home-based care) (*3*,*5*,*6*,*8*–*10*).

**Summary**

**What is already known about this topic?**

Each year, approximately 800,000 U.S. persons experience a stroke; outpatient stroke rehabilitation use among survivors helps improve outcomes and might reduce stroke recurrences.

**What is added by this report?**

In 2013, 31.2% of stroke survivors reported participation in outpatient stroke rehabilitation in 20 states and the District of Columbia. Reported use varied by demographic characteristics and by state. Among the four states reporting rehabilitation use for both 2013 (27.7%) and 2015 (35.5%), use increased significantly but remained suboptimal.

**What are the implications for public health practice?**

Implementing strategies that remove barriers and increase use of outpatient stroke rehabilitation among stroke survivors, with special focus among underserved populations, can increase positive health outcomes.

## Footnotes

* https://www.cdc.gov/brfss.

† The overall median response rates were 46.4% in 2013 and 47.2% in 2015 for all 50 states and territories with participants in the BRFSS.

§ Arizona, Arkansas, District of Columbia, Florida, Georgia, Hawaii, Iowa, Maine, Massachusetts, Minnesota, Mississippi, Missouri, Nebraska, North Carolina, North Dakota, Oklahoma, Oregon, South Carolina, Tennessee, Washington, and Wisconsin.

¶ Georgia, Iowa, Maine, and Oregon.

** *Healthy People 2020* Heart Disease and Stroke Objectives (HDS-23). https://www.healthypeople.gov/node/4588/data_details.

†† The Coverdell program works with health systems across funded recipient states to gather data and drive quality improvement in the prehospital, in-hospital, and posthospital care settings. https://www.cdc.gov/dhdsp/programs/stroke_registry.htm.
